# Neural induction drives body axis formation during embryogenesis, but a neural induction-like process drives tumorigenesis in postnatal animals

**DOI:** 10.3389/fcell.2023.1092667

**Published:** 2023-05-09

**Authors:** Ying Cao

**Affiliations:** ^1^ Shenzhen Research Institute of Nanjing University, Shenzhen, China; ^2^ MOE Key Laboratory of Model Animals for Disease Study, Model Animal Research Center of Medical School, Nanjing University, Nanjing, China; ^3^ Jiangsu Key Laboratory of Molecular Medicine of Medical School, Nanjing University, Nanjing, China

**Keywords:** neural induction, tumorigenesis, neural stemness, tumorigenicity, pluripotency, epithelial-mesenchymal transition, tumor microenvironment, body axis formation

## Abstract

Characterization of cancer cells and neural stem cells indicates that tumorigenicity and pluripotency are coupled cell properties determined by neural stemness, and tumorigenesis represents a process of progressive loss of original cell identity and gain of neural stemness. This reminds of a most fundamental process required for the development of the nervous system and body axis during embryogenesis, i.e., embryonic neural induction. Neural induction is that, in response to extracellular signals that are secreted by the Spemann-Mangold organizer in amphibians or the node in mammals and inhibit epidermal fate in ectoderm, the ectodermal cells lose their epidermal fate and assume the neural default fate and consequently, turn into neuroectodermal cells. They further differentiate into the nervous system and also some non-neural cells via interaction with adjacent tissues. Failure in neural induction leads to failure of embryogenesis, and ectopic neural induction due to ectopic organizer or node activity or activation of embryonic neural genes causes a formation of secondary body axis or a conjoined twin. During tumorigenesis, cells progressively lose their original cell identity and gain of neural stemness, and consequently, gain of tumorigenicity and pluripotency, due to various intra-/extracellular insults in cells of a postnatal animal. Tumorigenic cells can be induced to differentiation into normal cells and integrate into normal embryonic development within an embryo. However, they form tumors and cannot integrate into animal tissues/organs in a postnatal animal because of lack of embryonic inducing signals. Combination of studies of developmental and cancer biology indicates that neural induction drives embryogenesis in gastrulating embryos but a similar process drives tumorigenesis in a postnatal animal. Tumorigenicity is by nature the manifestation of aberrant occurrence of pluripotent state in a postnatal animal. Pluripotency and tumorigenicity are both but different manifestations of neural stemness in pre- and postnatal stages of animal life, respectively. Based on these findings, I discuss about some confusion in cancer research, propose to distinguish the causality and associations and discriminate causal and supporting factors involved in tumorigenesis, and suggest revisiting the focus of cancer research.

## 1 Introduction

Understanding the nature of cancer initiation and progression has experienced wild fluctuations, from the initial chaos of phenomenological description of cancer to the attempts of simplifying cancer regulation to single molecular events, and then followed by indefinite complexity of genetic and phenotypic heterogeneities and regulatory mechanisms (Weinberg, 2014). At present, about five million cancer literatures have been published. They showed that almost all aspects of biological research play a role in cancer, and nearly every gene is associated with cancer (de Magalhães, 2022). Some hypotheses/concepts have been proposed to generalize the rules behind the complexity of tumorigenesis, including notably the mutation theory, aneuploidy and chromosome instability, Warburg effect, epithelial-to-mesenchymal transition (EMT), *etc.* However, none of them can integrate the complexity of cancer because they explain tumorigenesis in some aspects but meet serious challenges in others (Soto and Sonnenschein, 2011; Paduch, 2015; Hanselmann and Welter, 2016). Notably, cancer was proposed as a disorder of developmental dynamics (Rubin, 1985), and the key signaling pathways involved in early embryogenesis also play important roles in cancers, for instances, the TGFβ, Wnt, FGF, Notch pathways. But how the complex process of embryogenesis is intrinsically linked with the complex process of tumorigenesis has remained a challenging question, particularly when considering that a normal embryogenesis needs a process of fusion of gametes. Cancer research is not the business only for biological and medical researchers. Astrophysicists proposed cancer as an atavistic reversion effect (Lineweaver et al., 2021). Moreover, quantum physics, the study on the discrete units of matter and energy throughout the Universe, is also suggested as the governing rule of cancer (Hameroff, 2004; Bordonaro, 2019; Laster et al., 2019). Despite the universality of physics, it is critical to find a particular link to integrate different data and phenomena, distinguish causality and associations, and figure out causal and supporting factors from the observations of cancer research. In the review, I will discuss my findings about the central role of neural stemness in cell tumorigenicity and pluripotent differentiation potential. This gives rise to a conceptual paradigm that might integrate different features of tumorigenesis. I propose that tumorigenesis might represent a distorted replay of neural induction and subsequent tissue differentiation during embryogenesis, a critical process required for neural development and normal body axis formation.

## 2 Embryonic neural induction and embryogenesis

How the nervous system is formed and body axis is established had been a primary focus of embryological study. Almost a century ago, a paramount work done by Spemann and Mangold showed that a small group of cells, the dorsal blastopore lip of a newt gastrula embryo, were capable of inducing formation of secondary body axis or a conjoined twin when they were transplanted to the ventral side of a host gastrula embryo. The secondary body axis, which contained neural tube, somites, pronephros and gut, was derived from the host, whereas the transplanted dorsal blastopore differentiated mostly into notochord (Spemann and Mangold, 1924; Spemann and Mangold, 2001). The dorsal lip was then named as the “Spemann-Mangold organizer”. An ectopic organizer induces formation of a conjoined twin containing neural tissues in a gastrula embryo. Vice versa, various studies showed that an embryo in the absence of organizer activity forms a “belly piece” only that contains no neural and dorsal structures (Spemann, 1938; Gerhart, 2001; De Robertis, 2009; Sosa et al., 2019).

Subsequent pursuit of the mechanisms underlying the induction of neural tissue and body axis by organizer was a failure in nearly 6 decades after the dorsal blastopore transplantation experiment (De Robertis, 2009). Revival of the research began until the end of 1980s because of a critical observation. Amphibian blastula ectodermal explants differentiated into only epidermal tissues when they were cultured in neutral saline *in vitro*. Surprisingly, when the explants were disaggregated into single cells for a few hours first and then re-aggregated again, ectodermal cells differentiated into neural cells exclusively (Godsave and Slack, 1989; Grunz and Tacke, 1989; Sato and Sargent, 1989), suggesting that removal of an extracellular signal is required for the ectoderm to adopt a neural fate, and neural fate might be the “default fate” of ectodermal cells. Afterwards, genes with localized expression in the organizer that showed the activity of neural induction and secondary axis formation were identified, including *noggin*, *chordin*, *cerberus*, *etc.* (Smith and Harland, 1992; Sasai et al., 1994; Bouwmeester et al., 1996; Harland, 2000; De Robertis and Kuroda, 2004; De Robertis, 2006). They encode for secreted proteins that inactivate the signaling pathways promoting epidermalization of ectoderm and ventralization of body axis, particularly the BMP signaling, via direct binding to the BMP ligands (Harland, 2000; De Robertis and Kuroda, 2004; De Robertis, 2006; Anderson and Stern, 2016). Moreover, inhibition of the receptor for activin, a BMP-related ligand of the TGF-β family, led to neuralization of ectoderm in absence of inducing factors and rescue of ventralized embryos (Hemmati-Brivanlou and Melton, 1994). In contrast to the initial aim for finding neural inducers, these lines of evidence demonstrate that the fate of ectoderm is neural by default and epidermal fate is induced, and the organizer promotes neural fate by inhibiting the signals that promote epidermal fate in ectoderm. This is the “neural default model” of ectoderm (Weinstein and Hemmati-Brivanlou, 1997; Muñoz-Sanjuán and Brivanlou, 2002).

Neural induction is a prerequisite for body axis formation, a process including differentiation of not only the nervous system, but also differentiation of mesodermal and endodermal tissues such as somite and gut. Neural induction means activation or upregulation of a spectrum of neural genes, forming a regulatory network defining neural plate, the undifferentiated precursor tissue of the central nervous system. Ectopic organizer activity led to the formation of conjoined embryos. Likewise, ectopic stimulation of genes with specific or enriched expression in embryonic neural cells, for example, the proto-oncogenes *eed*, *yy1*, *ski*, *egfr*, *erbb2*, *erbb4* in *Xenopus*, *gelsolin* and *msxB* in zebrafish, also causes formation of a partial secondary body axis or a conjoined twin, which contains both neural and non-neural tissues (Amaravadi et al., 1997; Satijn et al., 2001; Kanungo et al., 2003; Nie and Chang, 2006; Phillips et al., 2006). These genes are components of the regulatory network for embryonic neural cells. Their ectopic expression activates the neural regulatory network, leading to gain of neural fate in non-neural cells and formation of a second body axis. By contrast, disruption of embryonic neural genes causes defects in neural and axial differentiation in mouse embryos, ultimately leading to developmental arrest at early stages (Faust et al., 1995; Gassmann et al., 1995; Threadgill et al., 1995; Berk et al., 1997; Britsch et al., 1998; Donohoe et al., 1999). Moreover, neural plate specifies somite size and is required for somite development during early embryogenesis (Mariani et al., 2001). These results suggest the critical importance of embryonic neural genes and neural precursor cells in tissue differentiation. Neural stemness, refering to the collective property of primitive neural stem cells (NSCs), neural crest cells and adult NSCs, represents the general stemness, defining tumorigenicity and pluripotent differentiation potential, a notion that has been experimentally clarified (Clarke et al., 2000; Tropepe et al., 2001; Xu et al., 2021; Cao, 2022; Zhang et al., 2022). This unique property of neural stemness might be predestined by the evolutionary advantage of neural regulatory networks (Xu et al., 2021; Cao, 2022). Contribution of neural stemness to formation of the nervous system is self-evident. However, its contribution to non-neural differentiation is not. Neural crest cells are pluripotent and share regulatory network with cleavage stage embryos, differentiating into peripheral nervous system and many types of non-neural tissues/cells, such as melanocytes, skeletal and connective tissues, and medulla cells of the adrenal gland, *etc.* Locating between neural plate and epidermal ectoderm, neural crest is induced by interactions between neural plate and adjacent tissues (Selleck and Bronner-Fraser, 1995; Knecht and Bronner-Fraser, 2002; Buitrago-Delgado et al., 2015; Gilbert and Barresi, 2016; Pla and Monsoro-Burq, 2018). This means that pluripotency of neural crest cells is ultimately derived from neural plate cells. Neuromesodermal progenitors in the most posterior region of elongating embryos give rise to both spinal cord and paraxial mesoderm. These cells are presumably originated from anterior neural plate (Henrique et al., 2015; Sambasivan and Steventon, 2021). Therefore, neural induction generates neural precursor cells, which give rise to the differentiation of not only neural tissues, but non-neural tissues as well.

The studies above on neural induction and body axis formation were primarily performed with amphibian species, the newts and African clawed frog (*Xenopus laevis*). The functional homologue has been identified in all classes of vertebrates, such as fish, bird and mammalian embryos, which is known as the node. It exhibits the activity of neural induction and body axis formation similar to the organizer through conserved molecular mechanisms (Gerhart, 2001; Thisse and Thisse, 2015; Martinez Arias and Steventon, 2018). The neural default state exhibited by amphibian blastula ectodermal cells is also exhibited by mammalian embryonic stem cells (ESCs). Amphibian blastula ectodermal cells are the equivalents of ESCs, since they have the potential of differentiation into cell types of all three germ layers. ESCs are usually cultured in medium containing high-concentration of fetal bovine serum. They adopt a neural fate and turn into primitive NSCs when cultured in defined serum-free medium (Tropepe et al., 2001; Ying et al., 2003a; Smukler et al., 2006). In this cell fate transition, BMP signaling plays a critical role in inhibiting neural fate in ESCs (Ying et al., 2003b; Malaguti et al., 2013), a similar mechanism as observed in amphibian ectodermal cells.

In summary, either extracellular signals by the organizer or node or ectopic activation of neural genes in non-neural cells can cause the gain of neural fate in non-neural cells during gastrulation, i.e., neural induction, and leads to the formation of a secondary body axis or a conjoined twin. This is the paradigm for understanding how neural tissue and body axis are initiated to form during early embryogenesis. Neural induction is fundamental for neural development and embryogenesis. Nevertheless, aberrant occurrence of neural induction or a similar process might be associated with some most sophisticated pathological effects. It was proposed that a conjoined twin is formed when a secondary organizer-like activity is present in a gastrulating embryo, such as a human embryo (Levin, 1999). A neural induction-like process could also occur erroneously in cells of postnatal animals, which might be the general cause of tumorigenesis.

## 3 Neural induction-like process and tumorigenesis

As analyzed above, neural induction during embryogenesis means that non-neural (ectodermal) cells turn into neural precursor cells in response to either an extracellular signal inhibiting non-neural cell property or intracellular stimulation of embryonic neural genes. Ectopic neural induction during gastrulation causes formation of a conjoined twin. Neural stemness is characterized as the essential property of tumorigenic and pluripotent cells, and tumorigenesis could be envisioned a progressive loss of original cell identity and gain of neural stemness in postnatal cells (Southall et al., 2014; Cao, 2017; Li et al., 2020; Cao, 2022; Zhang et al., 2022), reflecting a neural induction-like effect. One obvious example for the comparability of tumorigenesis as a conjoined twin formation should be the teratocarcinomas/teratomas, which are composed of disorganized but histologically identifiable tissues or organs derived from all three germ layers, such as undifferentiated neural epithelial tissue and differentiated nerves from ectoderm, gut and glandular tissues from endoderm, and cartilaginous and muscle tissues from mesoderm. Teratocarcinomas/teratomas are usually found in the gonads, but they can also form in extragonadal tissues/organs (Chao et al., 2004; Gatcombe et al., 2004; Singhal and Jhavar, 2008; Agrawal et al., 2010). The mechanism underlying teratocarcinoma/teratoma formation has been rarely reported. In mouse, an inactivation mutation in the gene *Dnd1*, which encodes a master regulator for vertebrate germ cell development, causes progressive loss of germ cells and incidence of testicular teratoma (Youngren et al., 2005; Liu and Collodi, 2010). The pluripotent property of embryonal carcinoma cells (ECs) derived from teratocarcinoma was well characterized, which enlightened subsequent studies on pluripotency of ESCs (Andrews, 2002; Solter, 2006; Barbaric and Harrison, 2012). ECs form teratocarcinomas when transplanted into immunodeficient mouse hosts, and contribute to formation of chimeric embryos when transferred into blastocysts. EC pluripotent cell lines are characteristic of neural precursor or progenitor cells, they can be differentiated into neurons when treated with retinoic acid (Pleasure and Lee, 1993; Bain and Gottlieb, 1998; Negraes et al., 2012), a reagent inducing neuronal differentiation from NSCs. Neural stem/progenitor cells, which are tumorigenic and are capable of teratoma formation in immunodeficient mice (Xu et al., 2021), were isolated from teratocarcinoma/teratoma (Hasgekar et al., 1996; Kim et al., 2019). Therefore, ECs are characteristic of neural stemness, tumorigenicity and pluripotent differentiation potential. Like neural induction during embryonic development, teratocarcinomas/teratomas are the consequence of progressive loss of original cell identity and gain of neural stemness, which contributes to tumorigenicity and pluripotency (Xu et al., 2021; Cao, 2022; Zhang et al., 2022), in either germ or somatic cells.

A much broader range of tumorigenesis is the formation and progression of cancers that have been found in most postnatal tissues/organs. Growing evidence has shown that cancer cells, or generally tumorigenic cells, are characteristic of NSCs. Like NSCs and ECs, cells from different cancer types are capable of neuronal differentiation in response to inhibition of endogenous cancer promoting factors, which have a specific or enriched expression in embryonic neural cells during vertebrate embryogenesis, and play essential roles in maintaining neural stemness in both cancer cells and NSCs (Lu et al., 2017; Zhang et al., 2017; Lei et al., 2019; Chen et al., 2021; Zhang et al., 2022). In general, most (if not all) cancer-promoting genes are neural stemness genes or genes with enriched expression in embryonic neural cells during vertebrate embryogenesis. Cancer cells share both regulatory networks and cell property with NSCs or embryonic neural cells (Zhang et al., 2017; Cao, 2022). By contrast, non-neural tissue-specific genes and genes promoting differentiation are downregulated/silenced in cancer cells, and a substantial part of tumor suppressor genes are non-neural genes during embryogenesis (Cao, 2017; Zhang et al., 2017; Cao, 2022). This mode of expression change of cancer related genes reflects the progressive loss of original cell identity and gain of neural stemness, and consequently, the gain of tumorigenicity and pluripotency in cancer cells during tumorigenesis. For example, when the key muscle differentiation gene *Myod1* was knocked out in myoblast cells, the cells lost their myoblast identity and gain of neural stemness, tumorigenicity and pluripotent differentiation potential (Xu et al., 2021). Correspondingly, a recurrent mutation in *MYOD1* leads to a dominant-negative product that inhibits the function of wild-type protein in rhabdomyosarcomas (Kohsaka et al., 2014; Rekhi et al., 2016). Intestinal stem cells in *Drosophila* turned into a NSC-like state in response to the loss of a transcription repressor, and consequently, caused the formation of neuroendocrine tumor (Li et al., 2020). Neurons can also be dedifferentiated into an NSC-like state when a factor repressing NSC and cell cycle genes and maintaining neurons in a differentiated state was removed, leading to acquirement of tumorigenicity and tumor formation (Southall et al., 2014).

**FIGURE 1:**
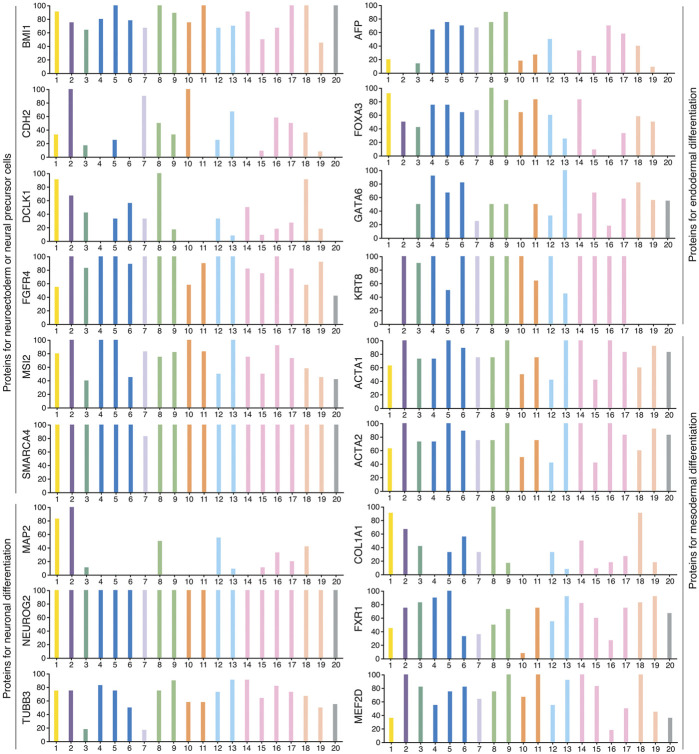
Typical marker proteins representing neuroectodermal or neural progenitor cells, neuronal cells, cells derived from endodermal differentiation, and cells derived from mesodermal differentiation during normal animal development were widely detected in various types of cancers. This indicates that tissue/cell types found in an animal body are also present in various cancers, suggesting cancers as degenerated embryoid bodies. For each cancer, color bars indicate the percentage of patients (maximum 12 patients) with high and medium protein expression level. Low or not detected protein expression results in a white bar. 1, Glioma; 2, Thyroid cancer; 3, Lung cancer; 4, Colorectal cancer; 5, Head and neck cancer; 6, Stomach cancer; 7, Liver cancer; 8, Carcinoid; 9, Pancreatic cancer; 10, Renal cancer; 11, Urothelial cancer; 12, Prostate cancer; 13, Testis cancer; 14, Breast cancer; 15, Cervical cancer; 16, Endometrial cancer; 17, Ovarian cancer; 18, Melanoma; 19, Skin cancer; 20, Lymphoma. Data are from the Human Protein Atlas (www.proteinatlas.org) (Uhlen et al., 2017).

Unlike teratocarcinomas/teratomas that are composed of a haphazard mixture of adult tissues and deformed organs, most other tumors usually do not contain well differentiated tissues and organs. Nevertheless, they are composed of cell types with distinct functional features and/or expression of tissue- or cell type-specific markers, indicating intratumor phenotypic heterogeneity. Two mainstream models are proposed to explain how phenotypic heterogeneity is generated. The clonal evolution model emphasizes that phenotypic heterogeneity is a result of genetic heterogeneity arising from Darwinian-like evolution. Nevertheless, how genetic heterogeneity causes phenotypic heterogeneity seems to be not understood at all and not testified experimentally. The cancer stem cell (CSC) model proposes that differentiation of CSCs generates phenotypic heterogeneity (Clevers, 2011; Marusyk et al., 2012; Beck and Blanpain, 2013; Burrell et al., 2013; Meacham and Morrison, 2013; McGranahan and Swanton, 2015; Quintanal-Villalonga et al., 2020), which has been validated in many studies. *In vitro* generated cells with CSC property can differentiate into cell types expressing neuronal, endothelial and muscle cell markers (Scaffidi and Misteli, 2011). CSCs of glioblastoma give rise to tumor endothelium and vascular pericytes, supporting tumor growth (Ricci-Vitiani et al., 2010; Wang et al., 2010; Cheng et al., 2013). CSCs of colorectal cancer revealed the capacity of multilineage differentiation (Vermeulen et al., 2008). A consensus conceptual framework could not be deduced from these studies about the nature of CSCs except that they can differentiate. It was not clear whether CSCs of different cancer types have the property of stem/progenitor cells of their respective tissues of cancer origin, or all CSCs might have a common property of stemness, or CSCs might be of “cancer-specific” nature that is not comparable to any known stem cell types. In fact, similar to ECs, cancer cells are also pluripotent because xenograft tumors formed by cancer cells show expression of markers of tissue/cell types derived from all three germ layers, for example, SOX1-expressing cells representing cells with neural stemness and derived from ectoderm, ACTA2-expressing cells derived from mesoderm, and AFP-expressing cells derived from endoderm (Xu et al., 2021; Cao, 2022; Zhang et al., 2022). These cells can be widely detected in different cancer types. Public databases show that a majority of transcripts and their protein products have low cancer specificity and are present in many cancer types (www.proteinatlas.org) (Uhlen et al., 2017). For example, BMI1, CDH2, DCLK1, FGFR4, MSI2, and SMARCA4 representing neural stemness; MAP2, NEUROG2, and TUBB3 representing neuronal differentiation; AFP, FOXA3, GATA6, and KRT8 representing endodermal tissue differentiation; and ACTA1, ACTA2, COL1A1, FXR1, and MEF2D representing mesodermal tissue differentiation (Figure 1). Noticeable is that ACTA2 and COL1A1 are also the markers of cancer-associated fibroblasts (CAFs). Therefore, different cancer types contain basic elements representing cell/tissue differentiation, similar to the case of embryogenesis. Cancer cells at different stage of tumorigenesis exhibit different degree of tumorigenicity. They are more similar to the cells of cancer origin and exhibit weak tumorigenicity at the beginning stage of tumorigenesis. Cancer cells at later stage are more dissimilar to the cells of cancer origin and show stronger tumorigenicity. Neural stemness and differentiation potential of cancer cells grow progressively and simultaneously with the progression of cancer, as suggested by a serial xenotransplantation assay of cancer cells (Zhang et al., 2022). Although cancer cells share the regulatory networks and cell property with neural stem or embryonic neural cells, some essential disparities still exist, including extensive defects in genes (differentiation genes in particular) and genome in cancer cells and the difference in the microenvironments with which cancer cells or embryonic neural cells communicate, leading to chaotic differentiation of cancer cells. In some cases, however, tissue/organ formation can be still observed, such as osteoid and bone formation in various cancers (Hoorweg et al., 1997; Goto et al., 2010; Dekkers et al., 2019; Kattepur et al., 2021; Tian et al., 2021). Cancers are degenerated forms of teratomas/teratocarcinomas or embryoid bodies, and tumorigenesis resembles an ectopic neural induction process and consequently embryonic tissue differentiation in postnatal animals. Cancer cells were demonstrated to induce secondary axis formation when transplanted into the appropriate position of a blastula stage of zebrafish embryo, implying a neural induction-like activity (Hendrix et al., 2007). Mutations in Wnt pathway that leads to stabilization and hence extra nuclear accumulation of *β*-catenin in cells are usually the cause of some cancers, e.g., colorectal cancer. Meanwhile, these mutations are able to cause the formation of a twinned mouse embryo (Brickman and Burdon, 2002), strengthening the intrinsic link between neural induction, conjoined twin formation and tumorigenesis. It is thus plausible that nearly every gene is associated with cancer (de Magalhães, 2022).

## 4 Epithelial-mesenchymal transition (EMT) means transition from a relatively known to an unknown cellular state, but EMT marker expression change reflects a neural induction-like program

EMT has been considered as a general rule governing malignant transformation of epithelial cells. EMT is described as a phenotypic change, in which a polarized epithelial cell loses its polarity and adhesion with neighboring cells, and assumes a mesenchymal phenotype with a motile property. At molecular level, the loss of epithelial phenotype is reflected by downregulation of the epithelial marker CDH1, and gain of mesenchymal phenotype is driven by a core set of EMT transcription factors, SNAI1, SNAI2, TWIST1, ZEB1, and ZEB2. EMT is believed to be the key driver of carcinogenesis and has been extensively investigated (Yang et al., 2020). Nevertheless, it has been heavily controversial because of its essential flaws, which were discussed in details previously (Cao, 2017). The main point is that both epithelial and mesenchymal states are highly heterogeneous among different tissues/organs and cannot be defined universally by a few “epithelial and mesenchymal” markers, and the meaning of “mesenchymal marker” expression change during malignant transformation of epithelial cells should be a misinterpretation (Cao, 2017). Moreover, some cancers are not originated from epithelial cells, but from mesenchymal cells, such as sarcomas. After 2 years of discussion, the EMT International Association (TEMTIA) in 2020 made a consensus statement about the guidelines and definitions of EMT research (Yang et al., 2020). The statement pointed out that the definition of classical EMT cannot reflect the complicated intermediate states between the binary switch from fully epithelial to fully mesenchymal state. Therefore, TEMTIA recommends that the definition of EMT should be more flexible and use “EMT plasticity” (EMP) to describe these intermediate states (Yang et al., 2020). The revised term can now smoothly fit all possible situations encountered during EMT research. However, the mechanisms underlying the boundless plasticity or how the plasticity is derived have not been understood at all. Furthermore, “while the characteristics of fully epithelial cells are relatively clearly defined, our current knowledge does not allow us to define the mesenchymal state with specific cellular characteristic or molecular markers that are universal end-products of all EMT programmes” (Yang et al., 2020). This means that EMT actually represents a transition from a relatively known to an unknown state. Many molecular mechanisms have been described for the regulation of EMT transcription factors in cancer or regulation of cancer progression by EMT transcription factors in numerous publications (Yang et al., 2020). But what the “mesenchymal state” is has remained unknown. In EMT or EMP, either the undefined plasticity (or “dynamics” in other literatures (Brabletz et al., 2021)) or the undefined mesenchymal state is used as a standard reference to define the characteristics of cancer cells. How this dilemma could fit for the regularly advocated logics of biological research, e.g., rigor, precision, and physiological relevance, is intriguing and worthy of pondering.

### 4.1 The alteration of EMT marker expression cannot be representative of the complex change during malignant transformation of epithelial cells

**FIGURE 2:**
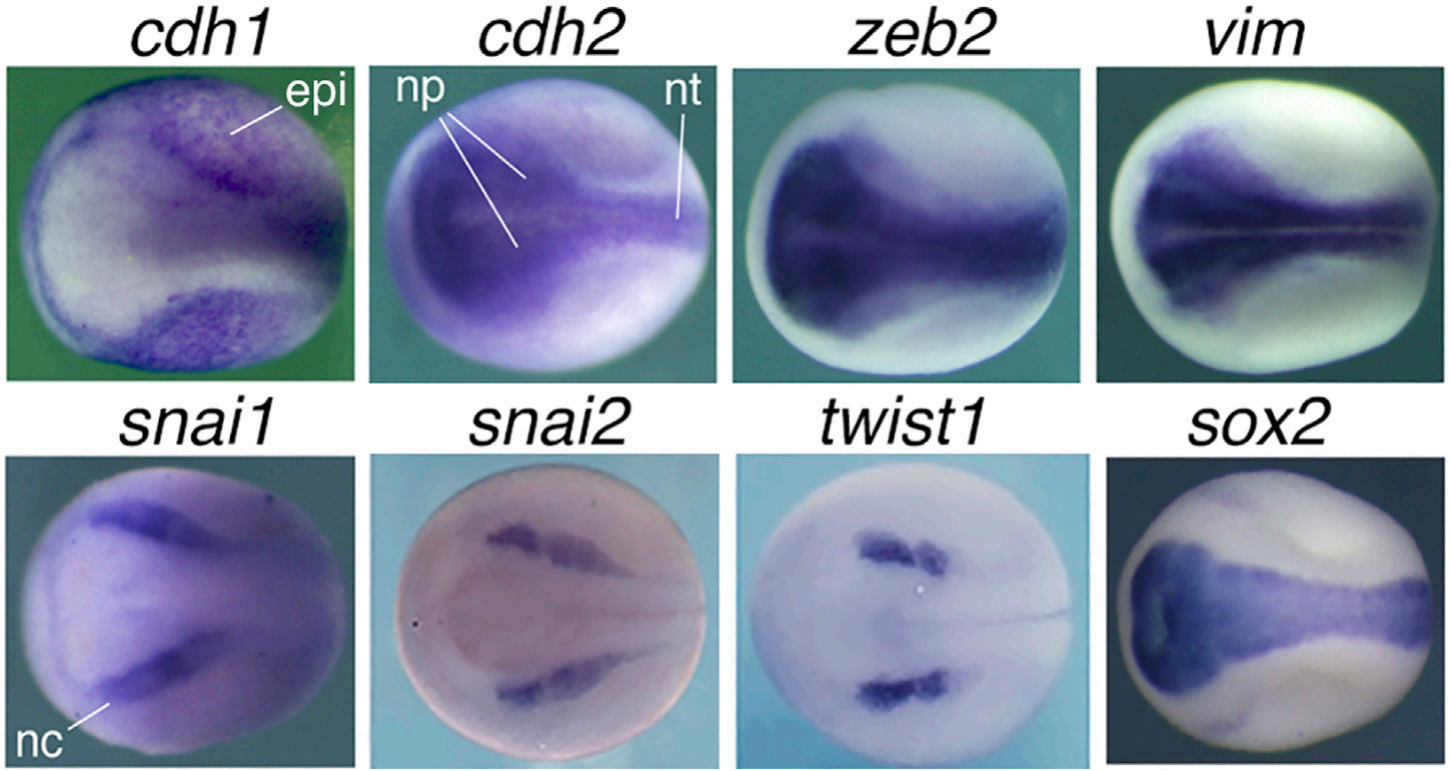
Expression patterns of “EMT” marker genes in neurula embryos of *Xenopus laevis*. Neural induction during gastrulation leads to formation of embryonic neural tissues in the subsequent developmental stage, during which the nervous system and various tissues/organs begin to form. Whole mount *in situ* hybridization revealed specific expression of *cdh1* in epidermis excluding the embryonic neural tissues, whereas *cdh2*, *zeb2* and *vim* are localized to neural plate, the precursor tissue of the central nervous system, and *snai1*, *snai2* and *twist1* are localized to neural crest, which give rise to the peripheral nervous system and many non-neural tissues. *sox2*, a marker gene for pluripotent stem cells and NSCs, is localized to neural plate and used as a control. Dorsal view is shown for each embryo with the anterior to the left. epi, epidermis; np, neural plate; nt, neural tube; nc, neural crest. Expression pattern data are from Zhang et al. (2017) and Wang et al. (2015) with permission from publisher.

Although epithelial cells of different tissues/organs express a same epithelial marker CDH1, they are derived from different lineages during development and execute distinct physiological functions in tissues/organs. Therefore, epithelial cells of different tissues/organs have different intrinsic regulatory networks. For example, epithelial cells of liver, which is differentiated from endoderm, must be different in function and regulatory network from those of kidney or skin, which are derived from mesoderm and ectoderm, respectively, during embryonic development. That is to say, besides the common epithelial property, epithelial cells of different tissues/organs are defined by tissue-specific genes/factors to perform tissue-specific functions. Downregulation of the epithelial marker is not the only event occurring during neoplastic transformation of epithelial cells. But rather, it is an associated or concomitant event among much more sophisticated changes: the progressive downregulation/silencing of tissue-specific and differentiation genes. This causes a dedifferentiation effect and loss of the property (including their epithelial state) and normal function of the original cells. On the other hand, many genes, including those encoding the “core EMT factors”, are upregulated/activated in cancer cells and play promoting roles during cancer initiation and progression. Unfortunately, this broad range of changes have not been considered by “EMT”. Cancer promoting genes may express and play diverse functions in normal embryonic and adult cells. However, their primary expression and function-a link between different cancer promoting genes-had been neglected. In fact, these genes belong to a same cellular context because most (if not all) of them are either neural stemness genes or genes with enriched expression in embryonic neural cells (Zhang et al., 2017). The typical “mesenchymal markers and transcription factors”, SNAI1, SNAI2, TWIST1, ZEB1, ZEB2, CDH2, VIM, are localized or at least enriched in embryonic neural cells, neuroepithelium, neural plate and neural crest (Wang et al., 2015; Zhang et al., 2017) (Figure 2), similar to most cancer promoting genes/factors. Upregulation of “core EMT factors” in cancer cells is concomitant with the upregulation of many other cancer promoting genes during cancer progression. Therefore, cancer cells share regulatory networks with NSCs or embryonic neural cells, but not other types of cells, and neural stemness is the determinant for cell tumorigenicity and pluripotent differentiation potential, a situation dictated by the evolutionary advantage of neural genes and neural state. In summary, malignant transformation of epithelial cells reflects the process of loss of original cell identity (including epithelial state) and gain of neural stemness (Cao, 2017; Zhang et al., 2017; Xu et al., 2021; Cao, 2022; Zhang et al., 2022).

### 4.2 The “mesenchymal state” shares little similarity with cancer cells in both cell features and regulatory networks

Furthermore, the “mesenchymal state” shares little similarity with cancer cells. Cancer cells are characteristic of rapid cell cycle and proliferation, stemness, dysregulated epigenetics and metabolism, cell motility, evasion of programmed cell death and immunosurveillance, resistance to therapies, plasticity, *etc.* (Hanahan and Weinberg, 2011; Bakir et al., 2020). There has been no evidence so far to demonstrate that any types of non-neural mesenchymal cells share these features and regulatory networks with cancer cells. Instead, these cell features are manifested by and the corresponding regulatory networks are enriched in NSCs or embryonic neural cells (Cao, 2017; Zhang et al., 2017; Chen et al., 2021; Xu et al., 2021; Cao, 2022; Zhang et al., 2022). The machineries for basic cell physiological functions, including cell cycle, ribosome biogenesis and protein translation, proteasome, spliceosome, epigenetic modifications, transcription, DNA replication, DNA damage and repair, and genes/factors promoting stemness, *etc.*, are all enriched in embryonic neural cells (Xu et al., 2021; Cao, 2022). They work concertedly together, but not alone, to define the property of high proliferation with pluripotent differentiation potential that serves as a fundamental cell property, i.e., neural stemness (Chen et al., 2021; Cao, 2022; Zhang et al., 2022). It is rather logical that all these basic machineries play active roles during tumorigenesis. Cancer cells always gain resistance to therapies, including immunotherapy, ultimately leading to a therapeutic failure. One of the most frequent mechanisms is the activation or upregulation of genes conferring resistance (Nussinov et al., 2017; Ramos et al., 2021). Actually, this is an intrinsic property of neural stemness. For example, EZH2 is involved in both chemoresistance and immunotherapy resistance (Hu et al., 2010; Kim et al., 2020; Reid et al., 2021). It is enriched in neural cells during vertebrate embryogenesis, maintains neural stemness, and is capable of dedifferentiating astrocytes into NSCs and confers stemness in cancer cells (Sher et al., 2011; Kim et al., 2013; Akizu et al., 2016; Zhang et al., 2017; Gorodetska et al., 2019; Lei et al., 2019). Cancer cells are characteristic of plasticity, which is usually explained as the consequence of EMP (Bakir et al., 2020; da Silva-Diz et al., 2018; Yuan et al., 2019). However, it should be kept in mind that genes in the regulatory networks of both cancer and embryonic neural cells are enriched in longer genes containing more exon/introns compared with those of non-neural cells (Sahakyan and Balasubramanian, 2016; Xu et al., 2021; Cao, 2022). Obviously, longer genes can serve as more flexible scaffold for regulatory signals for cell differentiation and functions, generate more splicing variants that contribute to phenotypic novelty and tissue identity (Baralle and Giudice, 2017; Bush et al., 2017). In agreement, the components of spliceosomes, the machinery responsible for alternative splicing, are expressed predominantly in embryonic neural cells and enriched in cancer cells, and promote cancers (Lee and Abdel-Wahab, 2016; Wang and Aifantis, 2020; Cao, 2022; Yamauchi et al., 2022). It was demonstrated recently that cell tumorigenicity and pluripotency are coupled properties unified by neural stemness. Synchronic enhancement of neural stemness, tumorigenicity and pluripotency is accompanied by increased level of proteins involved in translation, ribosome biogenesis and spliceosome assembly, *etc.*, and accordingly, increased events of alternative splicing in cancer cells (Zhang et al., 2022). It can be concluded that cancer cells are characteristic of neural stemness, but not mesenchymal state, in both cell features and regulatory networks.

### 4.3 The association between “EMT” or “EMP” and cancer cell features is within the context of neural induction-like program

Many studies have shown the association between “EMT” or “EMP” programs and cancer cell features, such as stemness, resistance to therapies, plasticity, *etc.* (Singh and Settleman, 2010; Brabletz et al., 2021). As shown in Figure 2, the epithelial marker gene *cdh1* is primarily expressed in non-neural ectoderm during vertebrate embryogenesis, while the typical “mesenchymal markers and transcription factors” are localized or enriched in embryonic neural cells, neuroepithelium, neural plate and neural crest. This means that the “core EMT transcription factors” are components of embryonic neural regulatory networks, similar to most cancer promoting factors. The neural specific expression readily establishes the correlation between the “core mesenchymal factors” and neural stemness, the ground state of pluripotency and tumorigenicity, and implies a far-fetched relationship with the “mesenchymal state”.

Contribution of “EMT” to cancer cell stemness was reported, but the underlying mechanisms have remained elusive (Lambert and Weinberg, 2021). Occasional studies demonstrated that stemness factors SOX2, BMI1 and OCT4 can be triggered by “EMT” factors ZEB1, SNAI1 and SNAI2 (Kurrey et al., 2009; Wellner et al., 2010; Mitra et al., 2018). Interestingly, Sox2, Bmi1, and Oct4 gene expression is localized to embryonic neural cells during neural induction and early neural development during vertebrate embryogenesis (Cao, 2022). Therefore, both “EMT factors” and stemness factors are components of the regulatory networks of a same cell type. “EMT” is associated with therapy resistance. One major mechanism for “EMT” associated therapy resistance is that “EMT factors” are able to induce transcription of genes encoding ABC transporters, such as ABCC4 and ABCC5 (Saxena et al., 2011). Resistance can also be enhanced by “EMT” via disruption of TP53 function, repression of tumor suppressor PTEN, or upregulation of pro-survival protein BCL-XL/BCL2L1 (Brabletz et al., 2021). The primary location of transcription of genes encoding ABC transporters, Tp53, Pten and Bcl2l1 is embryonic neural tissues (Zhang et al., 2017; Xu et al., 2021). Therefore, both “EMT factors” and therapy resistance factors are components of the regulatory networks of embryonic neural cells. The mechanisms underlying the contribution of “EMT” to cancer cell plasticity is also elusive. In the context of “EMT”, cancer cell plasticity is defined by the expression levels of “EMT markers”. A high level of epithelial marker expression in cancer cells indicates epithelial phenotype, whereas a high level of mesenchymal marker expression indicates “mesenchymal” phenotype. The intermediate states like “partial EMT”, “intermediate EMT”, *etc.*, are represented with hybrid expression of different levels of epithelial and mesenchymal markers, and hence, cancer cells can be grouped into distinct subtypes (Bakir et al., 2020; Brabletz et al., 2021; Esquer et al., 2021). Cancer cells with stronger “mesenchymal” phenotype are more strongly tumorigenic (Esquer et al., 2021). This is a logical dilemma because an undefined cellular state, the “mesenchymal state”, is used as a standard reference to define the phenotypic diversity of cancer cells. No matter it is a partial or a complete “mesenchymal state”, it is an unknown state. So far, it is not known mechanistically how the expression levels of “EMT markers” control cell plasticity. The situation will be changed when considering that the “mesenchymal markers” are actually integral components of the regulatory networks of NSCs or embryonic neural cells (Figure 2), i.e., neural stemness, which is a defined and plastic cell state. A key mechanism underlying cell plasticity is the enrichment of long genes and spliceosomes and hence alternative splicing in both cancer and embryonic neural cells (Sahakyan and Balasubramanian, 2016; Xu et al., 2021; Cao, 2022; Zhang et al., 2022). “EMT” is a concomitant event during carcinogenesis, but it has been misinterpreted as a causal or central factor. As having been proofed, it is difficult to find mechanisms and physiological relevance for a misinterpreted event. In summary, neural stemness, but not the undefinable mesenchymal state, is physiologically relevant with and integrates different characteristics of cancer cells and tumorigenesis (Xu et al., 2021; Cao, 2022).

## 5 Neural stemness of cancer cells and the tumor microenvironment: understanding the causality in cancer

Cancer research was dominated by a cancer cell-intrinsic view before 1980s since mutations in oncogenes and tumor suppressor genes were seemingly sufficient to determine cancer initiation and progression. This view could not explain smoothly the mechanisms governing cancer metastasis. Studies on tumor microenvironment (TME) and interactions between different cell types in the TME and between tumor and normal tissues might provide reasonable explanations, leading to a shift from the cancer cell-centric view to a tumor environment-centric view (Vogelstein and Kinzler, 1993; Maman and Witz, 2018; Garner and de Visser, 2020). Tumor-host interaction and crosstalks in TME is important for tumor growth and cancer progression. However, understanding the causal and supporting factors involved in the interactions and crosstalks is crucial not only for cancer biology, but also for the development of more efficient strategies of cancer therapy.

A tumor consists of heterogeneous populations of cells. A widely held view is that normal tissue cells infiltrate tumors or cancer cells acquire magic power to hijack normal cells, for example, nerves, immune cells, fibroblasts, blood vessels, *etc.*, and recruit them into TME to promote cancer progression (Maman and Witz, 2018; Saxena and Bhardwaj, 2018; Brandao et al., 2019; Cervantes-Villagrana et al., 2020; Lugano et al., 2020). The crosstalks between cancer cells and recruited normal tissue cells have been a major topic of study in cancer biology. How the cells in the TME are originated and how the functions of the cells are related with tumorigenesis have remained elusive.

### 5.1 The nerve-cancer crosstalk

The presence of nerves was observed in about one century ago and numerous subsequent studies have demonstrated that neural infiltration contributes to tumor progression and dissemination. High intratumor nerve intensity is correlated with poor prognosis and high recurrence across many cancer types (Jobling et al., 2015; Boilly et al., 2017; Cervantes-Villagrana et al., 2020; Reavis et al., 2020; Silverman et al., 2021). Promoting roles of nerves in cancer initiation and progression have been extensively investigated (Magnon et al., 2013; Hayakawa et al., 2017). It is believed that neural infiltration is achieved primarily by three ways: axonogenesis induced by neurotrophic factors (NGF, BDNF, GDNF) and axon-guidance molecules (Netrin-1, Ephrin B1) that are released from cancer cells, neural reprogramming or conversion of nerve types via extracellular vesicles derived from cancer cells, and neurogenesis as a result of differentiation of neural progenitor cells recruited by cancer cells (Silverman et al., 2021). Extracellular vesicle-induced neural reprogramming is dependent on Rab27A and Rab27B in cancer cells (Amit et al., 2020), which are required for extracellular vesicle release from cells (Ostrowski et al., 2010; Colombo et al., 2014). At least, Rab27A is predominantly expressed in neural tissues during vertebrate embryogenesis (Wuttke et al., 2016), suggesting that it is involved in neural development. Neurogenesis in tumors via recruitment of circulating progenitor cells from brain subventricular zone is rather provocative (Mauffrey et al., 2019). However, it needs to find out how neural progenitor cells break the brain-blood barrier and enter circulation, and what is the physiological significance of circulating neural progenitor cells. Under normal developmental processes and physiological conditions, neural cells are the primary source of neurotrophic factors and axon-guidance molecules. Either neural factors or extracellular vesicles released by cancer cells indicate that cancer cells have intrinsic features of neural cells, which communicate with and shape normal nerves. Studies have shown that cancer cells have the intrinsic potential of differentiation of neuronal cells (Lu et al., 2017; Zhang et al., 2017; Lei et al., 2019; Chen et al., 2021; Zhang et al., 2022), which compose of at least a part of nerves in a tumor (Reavis et al., 2020). Single-cell RNA-sequencing analyses also indicate that cancer cells are characteristic of neural cell state (Pascual et al., 2021; Venkataramani et al., 2022).

### 5.2 Cancer-associated fibroblasts (CAFs)

CAFs are one of the major cell types of tumor stroma and communicate with tumor cells and immune cells, thereby promoting or suppressing cancer progression (Kalluri and Zeisberg, 2006; Xouri and Christian, 2010; Alguacil-Núñez et al., 2018; Kobayashi et al., 2019; Miki et al., 2020; Miyai et al., 2020; Biffi and Tuveson, 2021). Very much similar to the “mesenchymal state”, CAFs are also an undefinable cell state because they are heterogeneous in marker expression, function and inter- and intra-tumoral phenotypes (Kobayashi et al., 2019; Biffi and Tuveson, 2021). CAFs are different from normal fibroblasts in both morphological and growth properties (Delinassios et al., 1983; Rønnov-Jessen et al., 1996). Little is known about the origin of CAFs in tumors, but a few possibilities were proposed (Xouri and Christian, 2010; Kobayashi et al., 2019). At the early stage of tumorigenesis, CAFs might be the remnant native fibroblasts from the tissue or organ of cancer origin. With the progression of tumorigenesis, new CAFs might be derived from transdifferentiation from a non-fibroblastic lineage, activation of existing resident fibroblasts, recruitment of circulating cells of a remote source (particularly the bone marrow mesenchymal stem cells), differentiation from cells with a stem or progenitor property, and even “EMT” (Kalluri and Zeisberg, 2006; Xouri and Christian, 2010; Kobayashi et al., 2019). Nevertheless, how different CAF types are related with their cellular origins has not been validated. For example, some studies considered local fibroblasts, bone marrow mesenchymal stem cells and pericytes as the origins of CAFs (Kalluri and Zeisberg, 2006; Hosaka et al., 2016; Koliaraki et al., 2017), but others showed that COL1A1^+^ and alpha-SMA^+^ CAFs are predominantly derived from local precursor cells rather than mesenchymal stem cells (Arina et al., 2016). Studies with mouse models and human patients showed that transplanted bone marrow cells are able to migrate to tumor sites and differentiate into some portion of CAFs in a tumor (Worthley et al., 2009; Quante et al., 2011). This does not mean that the circulating bone marrow progenitors are the only or main origin of CAFs. Cancer cells are pluripotent, they can differentiate into different cell types, including alpha-SMA^+^ cells. Cancer cells with stronger tumorigenicity and pluripotency can differentiate more efficiently, and alpha-SMA^+^ cells are more abundant in xenograft tumors formed by cancer cells with stronger tumorigenicity (Xu et al., 2021; Zhang et al., 2022). Stromal content is correlated with cancer progression and responses to therapy, and a high stromal content and a high level of CAFs in stroma is an indicator of poor patient prognosis (Huijbers et al., 2013; Sandberg et al., 2019; Vangangelt et al., 2020; Almangush et al., 2021; Hagenaars et al., 2021). These correlations also reflect that cancer cells with stronger tumorigenicity have stronger differentiation potential, i.e., tumorigenicity and pluripotency are coupled cell properties (Cao, 2022; Zhang et al., 2022), and CAFs might be at least partially derived from cancer cell differentiation.

### 5.3 Cancer-immune crosstalk

Immune cells, either innate (macrophages, neutrophils, dendritic cells, innate lymphoid cells, myeloid-derived suppressor cells, and natural killer (NK) cells) or adaptive (T and B Cells), are important constituents of tumor stroma (Maman and Witz, 2018; Hinshaw and Shevde, 2019). Cancer-immune crosstalk has been a mainstream study in cancer research, which sets up the basis for cancer immunotherapy. There exist many inconsistencies in the functions of immune cells in the TME. Tumor infiltrating T Cells exhibit both antitumor cytotoxicity (Vose et al., 1977; Brunner et al., 1981) and cancer-promoting activities (Vose and Moore, 1985; Wang et al., 2020a; Marcucci and Rumio, 2021). The M1 subset of macrophages exhibits antitumor activity, whereas the M2 subset plays a tumor-promoting role. The functions of dendritic cells, neutrophils, NK cells, *etc.*, all play both anti- and pro-tumor roles, depending on the subtypes of immune cells or on the types of cancers (Hinshaw and Shevde, 2019). Based on these understandings, researchers have made the best of anti-tumor function of immune cells and developed strategies of immunotherapy, particularly the engineered cytotoxic T Cells (CAR-T) and inhibitors of immune checkpoints. Immunotherapy has greatly revolutionized cancer therapy. However, the number of patients who can benefit from these therapies is still very limited. CAR-T therapy has shown high efficiencies in eliminating cancer cells of B Cell malignancies, but achieved little success in solid cancers due to high antigen heterogeneity in solid tumors, physical barriers preventing T Cell infiltration, and highly immunosuppressive TME that leads to T Cell exhaustion and dysfunction (De Bousser et al., 2021; Hou et al., 2021; Sterner and Sterner, 2021). Likewise, immune checkpoint inhibition achieves responses in only a minority of patients due to primary or intrinsic resistance of cancer cells (Sharma et al., 2017; Gide et al., 2018; Kalbasi and Ribas, 2020; Vitale et al., 2021), and sometimes cause even an adverse effect of hyperprogression (Champiat et al., 2018; Kamada et al., 2019; de Miguel and Calvo, 2020; Marcucci and Rumio, 2021). Meanwhile, therapy efficacy declines or disappears because cancer cells acquire resistance as therapy continues (Draghi et al., 2019; Gide et al., 2018; O'Donnell et al., 2019; Schoenfeld and Hellmann, 2020; Sharma et al., 2017). The mechanisms for resistance to immunotherapy are also a complicated issue and seem to be not more easily understood than understanding cancer itself. In general, resistance to immunotherapy is caused by insufficient tumor antigenicity due to the lack of tumor neoantigens, defects in transduction of anti-tumor immune response mediated by tumor-intrinsic IFNγ signaling, impaired antigen processing and presentation machinery, regulation by oncogenic signaling, and tumor dedifferentiation and stemness (Sharma et al., 2017; Draghi et al., 2019; Kalbasi and Ribas, 2020; Schoenfeld and Hellmann, 2020). These seemingly distinct mechanisms are actually interconnected together by the core feature of cancer cells, as exemplified in the following examples. MEX3B allows melanoma cells to evade tumour-specific T Cells via repression of HLA-A post-transcriptionally (Huang et al., 2018). Inhibition of CDK4/6 boosts antitumor immunity by increasing IL-2 production and tumor infiltration of T Cells (Kalbasi and Ribas, 2020). ADAR1 inhibition overcomes resistance to immune checkpoint blockade caused by inactivation of antigen presentation by tumour cells (Ishizuka et al., 2019). EZH2 plays an important role in immune checkpoint blockade resistance by regulating antigen presentation and antitumor immunity (Kim et al., 2020; Zhou et al., 2020). *β*-catenin activation impairs dendritic cell recruitment, promotes expression immune checkpoint genes, or represses T Cell genes in cancers (Spranger et al., 2015; Ruiz de Galarreta et al., 2019; Perry et al., 2020). SETDB1 promotes immune exclusion and resistance to immune checkpoint blockade in cancer cells by suppressing immunostimulatory genes (Griffin et al., 2021). Many studies established an association between “EMT” and tumor immunity, by showing that “EMT” is linked with upregulation of inhibitory checkpoint ligands, downregulation of tumor-associated antigens and inhibition of T Cell infiltration, *etc.* (Marcucci and Rumio, 2021). The intrinsic connection between these different mechanisms is that all these factors promoting immune therapy resistance are embryonic neural genes, and plays critical roles in neural development and tumorigenesis. This means that the resistance effect is concurrent with the gain or enhancement of tumorigenicity of cancer cells, i. e., neural stemness. In agreement, dedifferentiation and stemness of cancer cells is the key factor driving resistance to immunotherapy (Miao et al., 2019; Li and Stanger, 2020; Lei and Lee, 2021).

Cancer-initiating cells exhibit immune privilege, protecting cells from immune attack using the mechanisms mentioned above (Galassi et al., 2021). Accordingly, NSCs and embryonic stem cells, whose default state is NSCs, also exhibit immune privilege. They form teratomas in immunocompetent mice as the result of low expression of immune-related proteins, including MHC class I and II antigens, HLA-DR and co-stimulatory molecules (Magliocca et al., 2006; Itakura et al., 2017; Ozaki et al., 2017). In comparison, adult cells and non-neural cells are more susceptible to immune rejection (Drukker et al., 2006; Xu et al., 2021), suggesting that immunogenicity might be correlated with differentiation state.

It is believed that immune checkpoints promote tumorigenesis by regulating immune response of tumor cells to immune attack. Inhibitors of PD-1/PD-L1 have revolutionized cancer therapy. But inhibitors of subsequently identified immune checkpoints may have not achieve significant responses in patients. IDO1 functions in suppression of anti-tumor immunity by degrading tryptophan and producing a series of toxic kynurenine metabolites to promote immune evasion of tumors (Uyttenhove et al., 2003; Munn and Mellor, 2013), but the inhibitor of IDO1 failed in a phase 3 clinical trial (Long et al., 2019). The failure is in contrast to the mechanisms underlying IDO1 function in suppression of anti-tumor immunity. Emerging studies have elucidated some novel functions of the immune checkpoint PD-1 (PDCD1), which might be suggestive for the failures. PD-1 is primarily expressed in bone marrow and lymphoid tissues, and accordingly, enriched in activated T Cells, indicating a differentiated cell state. However, PD-1 expression was also detected in some subpopulations of cells of different cancers (Du et al., 2018; Wang et al., 2020b; Ieranò et al., 2022). Overexpressed PD-1/PD-L1 suppressed the viability, growth, proliferation and tumorigenicity of cancer cells, and inhibited tumor growth, whereas blocking cancer cell-intrinsic PD-1/PD-L1 generated an opposite effect. Therefore, in contrast to its tumor-promoting function in the context of immunity, cancer cell-intrinsic PD-1/PD-L1 works actually as a tumor suppressor (Du et al., 2018; Wang et al., 2020b; Ieranò et al., 2022), a finding that complicates cancer therapy using PD-1/PD-L1 blockade and the outcomes of patients in response to therapy. Mechanistically, inhibition of cancer cell-intrinsic PD-1/PD-L1 enhances AKT and ERK1/2 activity (Wang et al., 2020b). Genes for Akt (Akt1/2/3) and Erk1/2 (Mapk1/3) are all enriched in embryonic neural cells (Magdaleno et al., 2006; Xu et al., 2021), play critical roles in embryonic neural induction and neurodevelopment (Kuroda et al., 2005; Sittewelle and Monsoro-Burq, 2018), and promote tumorigenesis. Previous studies generalized that cancer promoting genes are mostly embryonic neural genes, which confer cells with neural stemness, and tumor suppressor genes are mainly non-neural or pro-differentiation genes, which suppress tumorigenicity by inhibiting neural stemness and confer cells with non-neural cell property (Cao, 2017; Zhang et al., 2017; Yang et al., 2021; Cao, 2022). Loss of PD-1 might cause a dedifferentiation effect in cancer cells and enhances their tumorigenicity. The novel function of PD-1/PD-L1 was identified mainly in *vitro* conditions. However, these studies indicate that it is not enough to understand the functions of immune checkpoints merely in the context of cancer-immune crosstalk.

### 5.4 Cancer cell-centric or tumor environment-centric?

There are some other cell/tissue types in tumors and more complicated crosstalks between multiple cell types have been described, e.g., the tumor-neuro-immune crosstalk (Kuol et al., 2018). As the mainstream viewpoint, cells in the TME are derived from normal tissues because of infiltration of circulating cells, invasion of cancer into normal tissues or hijacking of normal cells. In addition to these possible sources, it should be kept in mind that cancer cells are capable of pluripotent differentiation. The intra- and inter-tumoral phenotypic heterogeneity should be primarily resulted from differentiation of cancer cells. The cell types in a tumor might reflect a differentiation hierarchy of cancer cells, under the control of intra- and extracellular signals. Teratocarcinoma is a particular type of malignant tumors, which exhibit differentiation of histologically identifiable tissues/organs derived from all three germ layers, such as cartilage, bone tissues, neural epithelia, gut structures, *etc.* Formation of these tissues/organs in teratocarcinomas can be explained by the pluripotent differentiation potential of embryonal carcinoma cells and experimentally testified, but cannot be explained by hijacking of normal cells or cell infiltration. Osteoid and bone formation in primary tumors of various extraskeletal tissues, such as skin, breast, liver and rectum (Hoorweg et al., 1997; Goto et al., 2010; Dekkers et al., 2019; Kattepur et al., 2021; Tian et al., 2021) should be a consequence of cancer cell differentiation rather than recruitment of normal cells. It is possible that cancer cells can also differentiate into cells resembling immune cells, a topic remaining to be investigated.

Besides targeted and immune therapy of cancer, strategies of targeting TME components, e.g., vasculature, neuronal cells, CAFs, immune cells, *etc.*, have been emerging (Chung et al., 2010; Hao et al., 2021; Kaduri et al., 2021; Maia and Wiemann, 2021; Saw et al., 2022). TME is highly heterogeneous in context, functions and regulatory networks. For example, CD146^–^ CAFs inhibit ER expression in ER^+^ breast cancer cells, increasing resistance of tumor cells to Tamoxifen. By contrast, CD146^+^ CAFs maintain the expression of ER in ER^+^ breast cancer cells and sustain sensitivity to Tamoxifen (Brechbuhl et al., 2017), suggesting that similar subsets of CAFs can have distinct functions within different cancer subtypes. Targeting CAFs in TME via interference of TGF-β1/2, Furin, *etc.*, has been extensively investigated (Saw et al., 2022). These proteins are also expressed in cancer cells and play complex roles during tumorigenesis. TGF-β cytokines play dichotomous roles during tumor progression, they suppress cancer initiation but later promote cancer cell metastasis and immunoevasion (Yeh et al., 2019). Furin has been suggested as a potential target of therapy of some cancer types, but inhibition of Furin in some other cancers led to aggressive phenotypes (He et al., 2022). This means that inhibition of CAF-related proteins suppresses CAFs, and at the same time might also cause a promoting effect on cancer cells. Different immune cells and subpopulations of a same type of immune cells also generate contrasting effects on tumors, as mentioned above. Cancer cell intrinsic PD-1/PD-L1 being a tumor suppressor raises the question whether other immune checkpoints or TME molecular targets might also be expressed in cancer cells and serve as tumor suppressors. These complexities and inconsistencies make it a complicated issue to evaluate the efficacy of targeting TME (Maia and Wiemann, 2021). It needs to clarify how TME is derived from and how a gene/protein functions in cancer cells. A central role of neural stemness in tumorigenicity and pluripotent differentiation potential should be considered in both basic research and development of therapeutic strategies of cancer. A preliminary study showed that non-neural pro-differentiation factors inhibit cancer cell tumorigenicity effectively via conferring non-neural property in cancer cells and meanwhile inhibition of neural stemness and neural regulatory network (Yang et al., 2021).

## 6 Tumorigenicity as a manifestation of pluripotency in a postnatal animal

**FIGURE 3:**
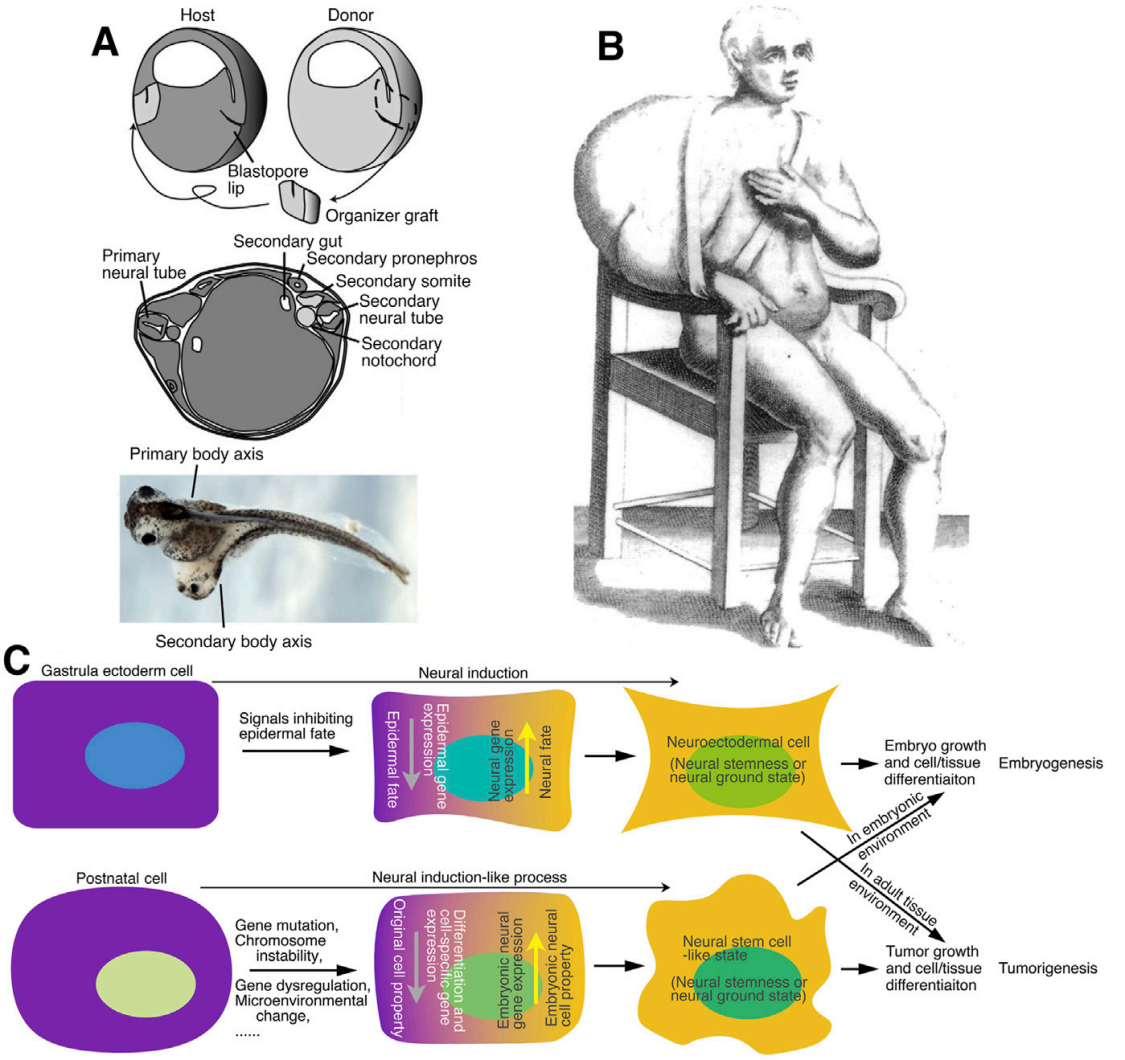
Neural induction, conjoined twin formation and tumorigenesis. **(A)** (Upper) A schematic illustration depicting the organizer graft transplantation experiment done by Spemann and Mangold, in which the organizer graft (the dorsal blastopore lip) of an early gastrula of a light-gray newt (*Triturus cristatus*) was grafted to the site opposite to the dorsal lip of an early gastrula of a dark-gray newt (*Triturus taeniatus*). (Middle) An illustration showing conjoined twin formation by organizer grafting. A section through the trunk of a conjoined twin embryo demonstrated that the light-gray graft contributed to the notochord, medial somite and floor plate of the secondary body axis, but the secondary neural tube, somites, pronephros, and archenteron cavity were induced from the dark-gray host embryo. (Lower) Conjoined twin formation of frog (*Xenopus laevis*) embryo by organizer grafting performed at early gastrula stage. **(B)** Tumor formation in human. The first clinical illustration of tumor, a large scapulohumeral tumor, most probably a sarcoma, in a book by a surgeon Marco Aurelio Severino published in 1,632 (Hajdu, 2011). **(C)** Neural induction or a similar process underlying both conjoined twin and tumor formation. During embryonic development, the organizer or node secretes proteins inhibiting epidermal fate of gastrula ectoderm, leading to the gain of neural fate in ectoderm and formation of neuroectoderm, a process known as “neural induction”. This is required not only for the differentiation of the nervous system but also for many non-neural tissues, such that the body axis of an embryo can form. Neural induction can occur ectopically during gastrulation, caused either by an ectopic organizer or node activity or by ectopic activation of embryonic neural genes, leading to the formation of secondary embryonic structures or a conjoined twin. This process might occur in any cell and at any time of animal life. Cells of a postnatal animal may suffer various extracellular (e.g., microenvironmental change) and/or intracellular (e.g., gene mutations) insults. If occasionally the insults cause activation of neural stemness regulatory network and/or downregulation/silencing of tissue-specific or differentiation genes/factors, cells progressively lose their original cell identity and gain of neural stemness or restore the neural ground state, similar to the neural induction process in gastrula ectodermal cells. The resulting cells can self-renew and differentiate into tissue/cell types of all three germ layers, resembling a defected process of embryonic development, that is, tumorigenesis. Tumorigenic cells (cancer cells, NSCs and embryonic pluripotent cells) are induced to differentiation into normal cells and integrate into normal embryonic development when they are placed in an embryonic environment, but they form tumors instead and cannot integrate into animal tissues/organs when they are in an environment in a postnatal animal because of lack of embryonic inducing signals. Therefore, tumorigenicity is a property of pluripotent cells manifested in a microenvironment of a postnatal animal. **(A)** is adapted from Harland (2008), **(B)** from Hajdu (2011), and **(C)** is from Zhang et al. (2022) with permission from publishers.

Tumorigenicity and pluripotency are key cell properties for tumorigenesis and embryogenesis. The proposal that neural stemness represents the ground state of and unifies cell tumorigenicity and pluripotency implies that tumorigenesis and embryogenesis are driven by a same general principle, the neural induction or a similar process. This provides fresh insights into the essence of tumorigenicity and tumorigenesis. A key property of ESCs is that they differentiate into various cells/tissues and contribute to chimeric formation when transplanted into blastocysts. Similar chimeric formation effects can be observed for NSCs (Clarke et al., 2000; Tropepe et al., 2001). Importantly, various types of cancer cells, including teratocarcinoma, leukemia, neuroblastoma and melanoma cells, can also contribute to chimeric formation or be induced to differentiate into different types of cells when transplanted into an embryo. The differentiated offspring cells are similar to host cells and not tumorigenic anymore (Brinster, 1974; Papaioannou et al., 1975; Illmensee and Mintz, 1976; Cooper and Pinkus, 1977; Gootwine et al., 1982; Podesta et al., 1984; Webb et al., 1984; Gerschenson et al., 1986; Wells and Miotto, 1986; Kulesa et al., 2006; Hendrix et al., 2007). A recent study highlights that the differentiation stage of cancer cells affects the consequence of tumorigenicity (Baggiolini et al., 2021). Moreover, transplantation of the nuclei of different cancer cells into enucleated oocytes led to development of normal embryos (King and DiBerardino, 1965; McKinnell et al., 1969; DiBerardino et al., 1983; Li et al., 2003; Hochedlinger et al., 2004), suggesting the pluripotent nature of cancer cells. Therefore, characterization of cancer cells and NSCs implies that variants of pluripotent state can be numerous and are present throughout the life of an animal from a pre-implantation blastocyst to adult stage. Chimeric formation indicates that pluripotent cells, including cancer cells, NSCs and embryonic pluripotent cells, can be induced to differentiate into normal cells in an embryonic milieu and integrated into the development of an embryo. Instead, they form embryoid structures that cannot integrate into normal differentiated tissues or organs in the absence of embryonic differentiation signals during tumorigenesis in a postnatal animal or under the skin of an immunodeficient mouse. The different behavior of pluripotent cells in embryonic milieu and in a postnatal animal suggests that tumorigenicity, a pathological property, is actually an aberrant manifestation of pluripotent state in a postnatal animal. These studies, together with a recent study, also suggest that differentiation of cancer cells induced by embryonic inducing factors could be an efficient therapeutic strategy of cancers (Yang et al., 2021). Different cancer cell features, such as rapid cell cycle and proliferation, stemness, dysregulated metabolism, dysregulated epigenetics, immunoevasion, *etc.*, are all integral constituents of neural stemness (Cao, 2017; Xu et al., 2021; Cao, 2022; Zhang et al., 2022). In conclusion, neural stemness and its regulatory network is the unique base on which an embryo or a tumor is built up. Figure 3 summarizes that neural induction, a process of loss of original cell identity and gain of neural stemness or restoration of neural ground state in cells, drives embryogenesis in gastrulating embryos but drives tumorigenesis in a postnatal animal. Tumorigenicity and pluripotency are both but different manifestations of the same cell property: neural stemness, in different stages of an animal life.

## 7 Perspectives

It is increasingly clear that malignant transformation of cells is a process of progressive loss of original cell identity and gain of the property of NSCs, a cellular property that determines both tumorigenicity and pluripotency, and unifies different malignant features, such as fast cell cycle/proliferation, motility, evasion of apoptosis and immune surveillance, dysregulated epigenetics and metabolism, therapy resistance, *etc.* (Cao, 2017; Xu et al., 2021; Cao, 2022; Zhang et al., 2022). Neural stemness is the prime cellular property that determines tumorigenesis. TME plays important roles during tumorigenesis and has been a main focus of cancer research. Nevertheless, how the cells in TME are derived and how the cells function in cancer have remained elusive. The TME of teratocarcinoma and the pluripotency of teratocarcinoma cells are inspiring. In addition to cells originating from the host, the pluripotent differentiation potential of cancer cells (or generally, tumorigenic cells) should be the prime factor to consider when deciphering TME and the functions of TME components in cancer.

As discussed above, great progresses have been achieved in cancer therapy, in particular the targeted and immune therapies. But many challenges persist, such as the limited number of patients who can benefit from the therapies and the insurmountable obstacle of therapy resistance. Many mechanistic studies have revealed that factors conferring cancer cells with therapy resistance are generally neural stemness or embryonic neural factors, suggesting that neural stemness is also the cell property responsible for resistance. Neural stemness is the key property of cancer cells, targeting neural stemness might be potentially an efficient strategy to suppress cancer and reduce resistance effect. That cancer cells can be induced to differentiate into non-tumorigenic cells in embryonic environment implies that inhibition of tumorigenesis might be achieved via differentiation, hence the loss of neural stemness in cancer cells. Differentiation therapy was suggested 50 years ago (Pierce and Wallace, 1971; Pierce, 1983). A variety of differentiation inducing agents, including neural growth factors, all trans retinoic acid, arsenic trioxide, butyric acid or cAMP, have been shown some degree of differentiation-inducing capability *in vitro* and/or *in vivo* experiments (de Thé, 2018). The best-known case of differentiation therapy is the treatment of acute promyelocytic leukemia with all-trans retinoic acid (Huang et al., 1988). But differentiation therapy has been not applied as widely as other therapies. If the key property of cancer cells is not understood, it will be difficult to find an appropriate approach to achieve efficient differentiation. The neural stemness property and pluripotency of cancer cells implies that they can be induced to differentiate by key differentiation factors, particularly those for embryonic tissue differentiation that can mimic embryonic differentiation environment. Indeed, preclinical studies have shown that pancreatic cancer cells can be induce to differentiate by a key pancreatic differentiation factor Ptf1a, and breast cancer cells can be induced to post-mitotic adipocytes, and leading to suppression of the cancers (Ishay-Ronen et al., 2019; Krah et al., 2019). My preliminary study also demonstrated that non-neural pro-differentiation factors inhibit tumorigenicity of cancer cells via inhibition of neural stemness (Cózar et al., 2021). Whether differentiation via targeting neural stemness can be widely applied for cancer therapy is worth further studies.
